# Construction and validation of the Oxford Neurodevelopment Assessment (OX-NDA) in 1-year-old Brazilian children

**DOI:** 10.1186/s12887-022-03794-1

**Published:** 2022-12-23

**Authors:** Michelle Fernandes, Diego Bassani, Elaine Albernaz, Andrea D. Bertoldi, Mariangela F. Silveira, Alicia Matijsevich, Luciana Anselmi, Suélen Cruz, Camila S. Halal, Luciana Tovo-Rodrigues, Gloria Isabel Nino Cruz, Deepa Metgud, Ina S. Santos

**Affiliations:** 1 MRC Lifecourse Epidemiology Centre and Human Development & Health Academic Unit, Faculty of Medicine, University of Southampton, Southampton, UK; 2 Nuffield Department of Women’s and Reproductive Health, John Radcliffe Hospital, University of Oxford, Oxford, UK; 3 Oxford Maternal and Perinatal Health Institute, Green Templeton College, University of Oxford, Oxford, UK; 4 Dalla Lana School of Public Health & Faculty of Medicine, Department of Paediatrics, University of Toronto, Toronto, Canada; 5 Centre for Global Child Health, The Hospital for Sick Children, Toronto, Canada; 6 Maternal and Child Department, Faculty of Medicine, Federal University of Pelotas, Pelotas, RS Brazil; 7 Post-graduate Program in Epidemiology, Federal University of Pelotas, Pelotas, RS Brazil; 8 Departamento de Medicina Preventiva, Faculdade de Medicina FMUSP, Universidade de São Paulo, São Paulo, SP Brazil; 9 Conceição Hospital Group, Hospital Criança Conceição, Porto Alegre, RS Brazil; 10 Department of Paediatric Physiotherapy, KLE Institute of Physiotherapy, JN Medical College, KLE University, Belagavi, India; 11 Post-graduate Program in Pediatrics and Child Health, Pontifical Catholic University of Rio Grande do Sul (PUCRS), Porto Alegre, RS Brazil

**Keywords:** Neurodevelopment, Early child development, Infant development, Developmental delay, OX-NDA, 2015 Pelotas birth cohort study

## Abstract

**Background:**

Over 250 million children under 5 years, globally, are at risk of developmental delay. Interventions during the first 2 years of life have enduring positive effects if children at risk are identified, using standardized assessments, within this window. However, identifying developmental delay during infancy is challenging and there are limited infant development assessments suitable for use in low- and middle-income (LMIC) settings. Here, we describe a new tool, the Oxford Neurodevelopment Assessment (OX-NDA), measuring cognition, language, motor, and behaviour, outcomes in 1-year-old children. We present the results of its evaluation against the Bayley Scales of Infant Development IIIrd edition (BSID-III) and its psychometric properties.

**Methods:**

Sixteen international tools measuring infant development were analysed to inform the OX-NDA’s construction. Its agreement with the BSID-III, for cognitive, motor and language domains, was evaluated using intra-class correlations (ICCs, for absolute agreement), Bland-Altman analyses (for bias and limits of agreement), and sensitivity and specificity analyses (for accuracy) in 104 Brazilian children, aged 12 months (SD 8.4 days), recruited from the 2015 Pelotas Birth Cohort Study. Behaviour was not evaluated, as the BSID-III’s adaptive behaviour scale was not included in the cohort’s protocol. Cohen’s kappas and Cronbach’s alphas were calculated to determine the OX-NDA’s reliability and internal consistency respectively.

**Results:**

Agreement was moderate for cognition and motor outcomes (ICCs 0.63 and 0.68, *p* < 0.001) and low for language outcomes (ICC 0.30, *p* < 0.04). Bland-Altman analysis showed little to no bias between measures across domains. The OX-NDA’s sensitivity and specificity for predicting moderate-to-severe delay on the BSID-III was 76, 73 and 43% and 75, 80 and 33% for cognition, motor and language outcomes, respectively. Inter-rater (*k* = 0.80-0.96) and test-rest (*k* = 0.85-0.94) reliability was high for all domains. Administration time was < 20 minutes.

**Conclusion:**

The OX-NDA shows moderate agreement with the BSID-III for identifying infants at risk of cognitive and motor delay; agreement was low for language delay. It is a rapid, low-cost assessment constructed specifically for use in LMIC populations. Further work is needed to evaluate its use (i) across domains in populations beyond Brazil and (ii) to identify language delays in Brazilian children.

**Supplementary Information:**

The online version contains supplementary material available at 10.1186/s12887-022-03794-1.

## Background

In 2015, Early Child Development (ECD) was, for the first time, included in the Sustainable Development Goals as indicator 4.2.1: “the proportion of children under 5 years of age who are developmentally on track in health, learning and psychosocial well-being, by sex” [1]. Nevertheless, over 250 million children, globally, are at risk of not achieving their developmental potential due to poverty and stunting alone [2]. A disadvantaged start in life limits children’s abilities to benefit from education leading to lower productivity and social consequences that affect not only present but also future generations [3–7]. Moreover, brain stimulation interventions during the first 2 years of life have been to have enduring positive effects if children at risk are identified [3], using standardized assessments, and receive such interventions, within this window. One of the key challenges in the ECD landscape, therefore, is the early and accurate identification of children at risk, using standardized methodologies, to target interventions and to enable cross-population comparisons [5, 7, 8].

Many developmental batteries assess ECD outcomes during the first year of life [9] (Additional file 1: Table S1). However, only a few can be administered reliably by non-specialists and be applied across population groups from high-, middle- and low-income settings at relatively low costs [9–12]. Between 2013 and 2018, a WHO commissioned team identified only 3 initiatives that attempted to address these technical and logistical challenges [12]: (i) the WHO Gross Motor Milestones [13, 14], (ii) the Ages and Stages Questionnaires (ASQ) [15], and (iii) the Guide for Monitoring Child Development (GMCD) [16, 17]. Two of these (the ASQ and GMCD) were caregiver-reports; and the WHO milestones, although observer-rated, focussed exclusively on measuring gross motor skills.

Previous ECD research has shown that a mixed-methodology approach, combining direct-assessments with observer- and caregiver-reports, provides advantages over either method alone. This approach has been successfully applied in several developmental tests including the Batelle Developmental Inventory (BDI) [18, 19], the Test de Aprendizaje y Desarrollo Infantil (TADI) [20] and the INTERGROWTH-21st Neurodevelopment Assessment (INTER-NDA; www.inter-nda.com) [21]. The latter is a rapid, standardized, psychometrically valid 37-item international ECD instrument measuring cognition, fine and gross motor, language and behaviour outcomes in children aged 22 to 30 months [21, 22]. It can be administered reliably by non-specialists to identify children at risk of delay, and its norms are international ECD standards constructed according to the WHO’s prescriptive approach [23].

The adoption of the INTER-NDA - for the identification of children at risk of delay and for cross-population comparisons - by the clinicians and researchers, internationally, prompted demand for a tool, similar in conceptual and technical constructs, for the comprehensive and reliable assessment of outcomes in younger children [24]. The objective of this study is (i) to describe the rationale and methodology leading to the construction of a novel infant development assessment meeting the aforementioned specifications (the Oxford Neurodevelopment Assessment; OX-NDA) and (ii) to evaluate its performance against the Bayley Scales of Infant Development III edition (BSID-III) [25]. The specific aims of the current study were to: (i) examine agreement between OX-NDA and BSID-III domain scores; (ii) evaluate the OX-NDA’s sensitivity and specificity in predicting moderate-to-severe developmental delay on the BSID-III; and (iii) determine the OX-NDA’s internal consistency and rater reliability.

## Methods

### Participants and procedures

Infants enrolled in the 2015 Pelotas Birth Cohort Study [26], were consecutively recruited to participate in the OX-NDA validation study. Children with known severe hearing or vision impairments, and non-singleton children, were excluded from participation.

The 2015 Pelotas Birth Cohort is a large epidemiological study of child health, growth and development in the city of Pelotas in Southern Brazil. The cohort included 4275 newborns born between 1 January and 31 December 2015. Mothers and children were assessed at birth, and at 3, 12, 24 and 48 months post birth. Details of the cohort have been previously published [26]. The cohort is registered at clinicaltrials.gov [NCT03271723].

Participating children were evaluated on the OX-NDA and the BSID-III between 10 and 14 months by separate assessors, blinded to each other results. Assessments were conducted in Brazilian Portuguese in the children’s homes, in the presence of a primary caregiver over two consecutive days. In half the sample, the BSID-III was administered first; in the remaining sample, the OX-NDA was administered first. We did not evaluate children on both instruments in a single sitting to avoid children’s underperformance due to fatigue from repeated testing and because of the BSID-III’s long administration time (60-90 minutes). The reliability of the OX-NDA was determined for four non-specialist field assessors across 10 assessments.

**Fig. 1:**
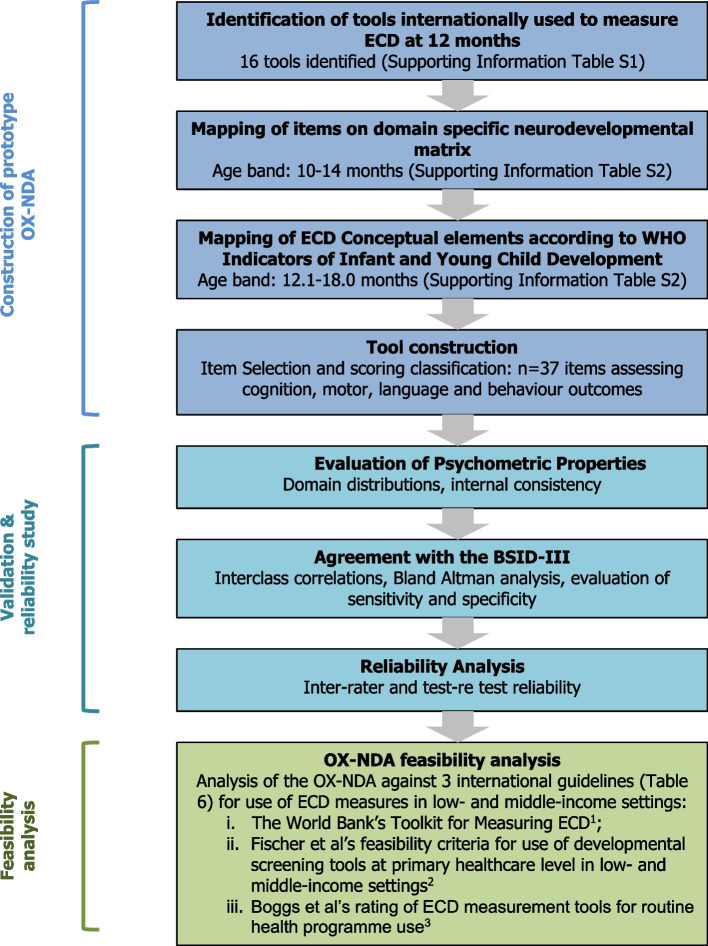
Flow chart of OX-NDA development and study process

The design, development and evaluation process of the OX-NDA is summarised in Fig. 1.

#### Measures

*The BSID-III:* The BSID-III measures cognition, language, motor, social-emotional, and adaptive behaviour outcomes in children aged 1-42 months [25, 27]. The thresholds for moderate and severe delay on its composite scores (Mean 100, SD 15) are 71-85 and ≤ 70, respectively [27]. Its latest norms were developed from 1700 US children recruited in 2004 and selected to match the 2000 United States census population of children [25]. The cost of the BSID-III kit, at the time of writing this paper, is GBP 1521.64, the cost per form per child is GBP 4.89 [27]. In this study, the adaptive behaviour subscale of the BSID-III was not included in the cohort’s protocol.

**Fig. 2:**
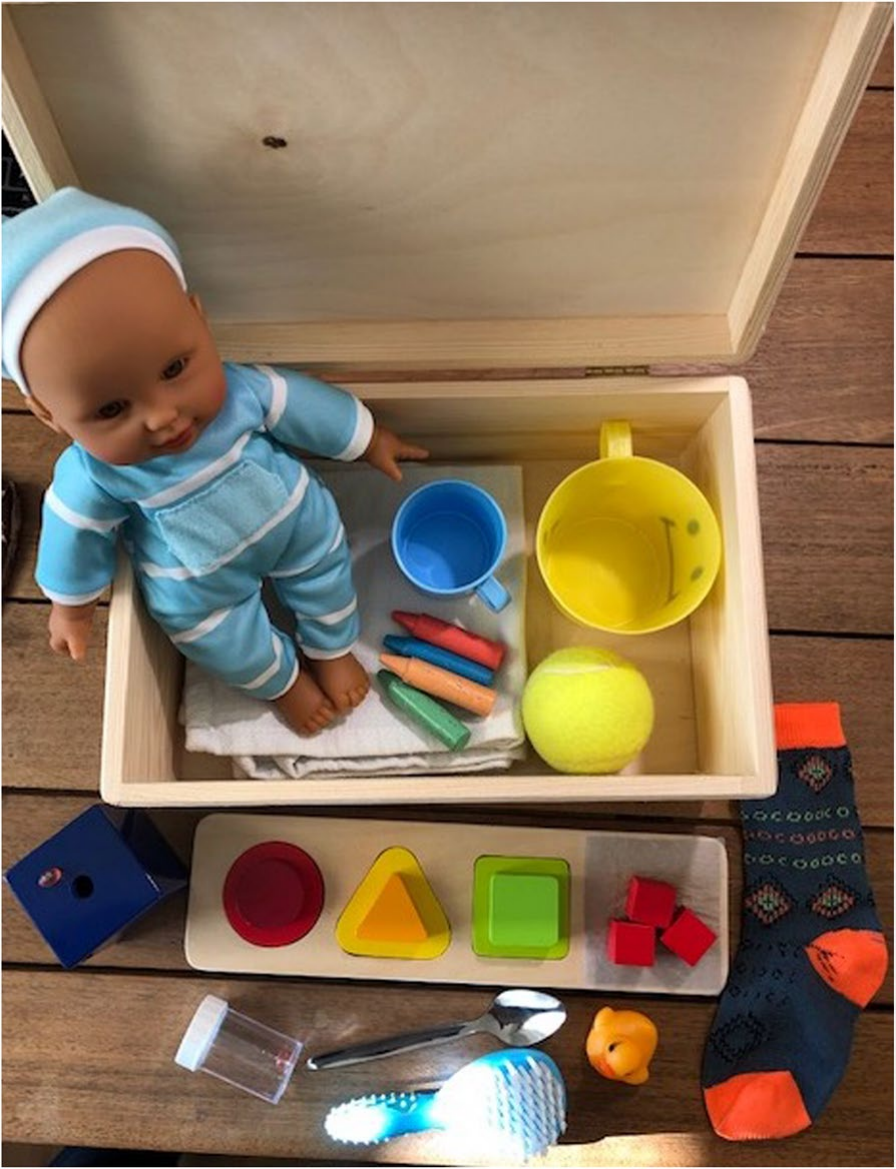
OX-NDA Kit

*The OX-NDA:* The OX-NDA is a novel multi-dimensional ECD assessment for children aged 10-14 months [24] consisting of 37 items grouped into cognition, motor, language, and positive and negative behaviour domains (Table 1). Of these items, 21 are directly assessed, 13 are concurrently observed and 3 are caregiver reported. Item scores range from 4 (highest) to 1 (lowest) with ‘unable to assess’ being scored as ‘X’: items scored ‘unable to assess’ are excluded from the calculation of mean domain scores. Its kit (Fig. 2) costs GBP 100.00 in the UK, but sourcing items locally can substantially reduce costs. There is no fee per child for use. The OX-NDA items were created in English and were translated into Brazilian Portuguese according to the WHO Mental Health Initiative translation guidelines which includes processes of translation, back translation and cultural customization [28].

**Table 1:** OX-NDA Domain Classification, Scoring Formulae and Interpretation

OX-NDA domain	Number of items contributing to domain	Range for item scores	Constituent item numbers for domain	Raw domain score estimation	Scaling formula for conversion of raw domain scores to standardized scores (range 0-100)	Interpretation of domain score
Cognition	15	1 - 4	1, 2, 3, 4, 5, 6, 7, 8, 9, 10, 11, 13, 14, 15, 16	Mean of constituent item scores	((x – 1) / 3)) * 100	Higher score reflects better performance
Motor	8	1 - 4	12, 17, 18, 22, 23, 24, 25, 26	Mean of constituent item scores	((x – 1) / 3)) * 100	Higher score reflects better performance
Language	7	1 - 4	19, 20, 21, 27, 28, 29, 30	Mean of constituent item scores	((x – 1) / 3)) * 100	Higher score reflects better performance
Positive behaviour	5	1 - 3	31,32,33,34,35	Mean of constituent item scores	((x – 1) / 2)) * 100	Higher score reflects better performance
Negative behaviour	2	1 - 3	36,37	Mean of constituent item scores	((x – 1) / 2)) * 100	Lower score reflects better performance

The construction of the OX-NDA involved five stages:

1. A landscape analysis of widely used tools measuring child development at 12 months across populations (Additional file 1: Table S1)
2. An analysis mapping the conceptual basis of the constituent items within each tool onto a ‘neurodevelopment domain’ matrix (Additional file 2: Table S2).
3. A second analysis mapping these items onto the WHO Indicators of Infant and Young Child Development (IYCD) ECD elements for 6.1-18 month age bands [12] (Additional file 2: Table S2).
4. Selection, by a panel of regional and international experts in ECD measurement, of the most appropriate ECD elements (*n* = 41) for inclusion in the prototype OX-NDA and construction of a 5-point performance reporting scale for each item (Table 1), similar to that of the INTER-NDA, to objectively measure a child’s level of achievement for the respective item (Additional file 3: Table S3).
5. Initial piloting and assessment of internal consistency (Table 3) resulted in excluding 4 items. The OX-NDA format for further psychometric testing and validation against the BSID-III therefore consisted of 37 items (Additional file 3: Table S3).

### Sample size and statistical analysis

Statistical analysis was conducted in Stata 15 software (Stata Corp., College Station, TX, USA). Accounting for attrition, a sample size of 104 was estimated to detected associations at a confidence level of 95% and significance level of < 0.05. Raw mean OX-NDA domain scores were calculated and converted to standardised scaled scores (Mean 50, SD 25, range 0 to 100; Table 1), and distributions explored. The following analyses were carried out:

1. Internal consistency: was determined for OX-NDA domain scores using Cronbach’s alphas. Values ≥0.7 were considered “good” [29, 30].
2. Agreement between the OX-NDA and BSID-III: was evaluated using four statistical methods, as recommended by Lee [31] and Bland and Altman [32]:

i. Continuous correlations (Pearson) to determine whether children who scored high on the OX-NDA also scored high on the BSID-III;
ii. Single measure intra-class correlation coefficients (ICCs) for absolute agreement between each domain, using a two-way mixed effects model (to quantify the strength of the association between OX-NDA and BSID-III scores);
iii. Bias and limits of agreement statistics; and
iv. Bland-Altman plots to identify whether the OX-NDA scores differed systematically across different levels of the BSID-III.

3. Sensitivity and Specificity Analyses: OX-NDA scores were compared between children scoring ≥85 (to assess OX-NDA sensitivity) and < 85 on BSID-III composites (to assess specificity). Cut-offs with highest sensitivity and specificity for each OX-NDA domain were determined using Receiver Operating Characteristics (ROC) analyses. Positive and negative predictive values and positive and negative likelihood ratios for each OX-NDA cut-off threshold were calculated.
4. Inter-rater and Test-re test Reliability: was determined using Cohen’s kappa coefficients [33].

### Ethics

Our study was approved by the Research Ethics Committee of the Medical School (No 1.400.585) and the School of Physical Education at the Federal University of Pelotas, Brazil (CAAE registration number: 26746414.5.0000.5313). Mothers provided written informed consent on behalf of their children.

## Results

### Sample characteristics

Of the cohort’s 4018 children followed-up at 12 months, a sub-sample of 104 was randomly selected to participate in the OX-NDA study. Other than age at weaning, there were no significant differences in socio-demographic and health characteristics between the OX-NDA sample and the cohort (Table 2). Children were assessed at a mean age of 367.6 (SD 8.4) days, 55 (53%) were male, and 84 (80.7%) were born at or near term. Mean birth weight was 3.2 (SD 0.5) kg. At the time of the assessment, children weighed 10.1 (SD 1.2) kg (corresponding to the 75th-85th centiles for girls and 50th-75th centiles for boys on the age-specific WHO child growth standards), and measured 75.1 (SD 3.1) cm in length (corresponding to the 50th-75th WHO centiles for girls and 25th-50th centiles for boys). The mean OX-NDA assessment time was 20.0 (SD 5.0) minutes.

**Table 2:** Sample Characteristics

	Children participating in the OX-NDA evaluation study (*n* = 104) Mean (SD) or number (%)	Children in the Pelotas 2015 Birth Cohort not participating in the OX-NDA evaluation study (*n* = 3914) Mean (SD) or number (%)	*p* value
Socio-demographic, prenatal and perinatal characteristics			
Maternal age at recruitment, years	27.1 (6.6)	27.5 (6.2)	0.51
Monthly family income	3585.6 (5667.7)	3010.9 (4377.3)	0.55
Duration of mother’s formal education, years	10.1 (4.0)	10.7 (4.0)	0.09
Maternal employment status	49 (47.1%)	1878 (47.9%)	0.06
Maternal infections (including HIV, rubella, syphilis, hepatitis B, CMV, toxoplasmosis, tuberculosis and malaria)	10.0 (11.6%)	348 (9.6%)	0.49
Maternal substance abuse (including alcohol) and smoking	20 (19.2%)	582 (16.1%)	0.18
Maternal prenatal anxiety and depression/mental stress	15 (14.4%)	402 (11.1%)	0.26
Maternal preeclampsia and eclampsia	10 (9.6%)	227 (6.3%)	0.10
Perinatal and Neonatal characteristics			
Gestational age at delivery, weeks	38.7 (2.0)	38.4 (2.4)	0.34
Birth weight, kg	3.2 (0.5)	3.2 (0.5)	0.39
Birth length, cm	48.3 (2.6)	40.2 (3.2)	0.64
Head circumference at birth, cm	33.9 (1.5)	33.9 (2.9)	0.94
Apgar score at 5 min	9.5 (0.6)	9.7 (4.3)	0.13
Boys	55 (53.0%)	1848 (51.1%)	0.72
Postnatal characteristics			
Only child	51 (49.0%)	1905 (48.7%)	0.95
Mean age of 12-month / OX-NDA assessment (days)	367.6 (8.4)	–	–
Significant morbidity during the first year of life^1^	4 (3.8%)	153 (5.1%)	0.73
Weight at 1 year, kg	10.1 (1.2)	9.9 (1.4)	0.28
Length at 1 year, cm	75.1 (3.1)	75.0 (3.0)	0.63
Immunized for age	90 (100%)	2955 (99.7%)	0.60
Age at which infant was weaned, months	5.7 (3.4)	4.1 (3.8)	*0.001
BSID-III assessment conducted before OX-NDA	52 (50%)	–	–
Duration between OX-NDA and BSID-III assessments at 1 year, day	1 (0)	–	–

*p < 0.05

*OX-NDA* The Oxford Neurodevelopment Assessment

^1^Signficant morbidity during the first year of life is defined as any life threatening or life altering condition, requiring prolonged hospitalization and/or treatment, such as epilepsy, metabolic disorders, endocrinological disorders, haematological disorders including haemophilia, oncological diagnosis, any conditions requiring surgery, congenital cardiac conditions, prolonged ventilation included home-based ventilatory support and/or neurological conditions requiring prolonged treatment and/or surveillance

### Internal consistency

For the final OX-NDA (consisting of 37 items), Cronbach’s alpha values (Table 3) were satisfactory (≥0.70) for the cognition, motor and positive behaviour; acceptable (0.40) for language, and weak (< 0.4) for negative behaviour [30].

**Table 3:** Internal consistencies of OX-NDA domain scores

OX-NDA Domain	Prototype OX-NDA		Final OX-NDA	
	N (items)	Cronbach’s alphas	N (items)	Cronbach’s alphas
Cognition	15	0.71	15	0.71
Motor	8	0.70	8	0.70
Language	8	0.30	7	0.40
Executive Function	3	0.42	0	–
Positive behaviour	5	0.72	5	0.72
Negative behaviour	2	0.22	2	0.22

*OX-NDA* The Oxford Neurodevelopment Assessment

### Concurrent validity between the OX-NDA and BSID-III

Strong positive correlations were observed between cognition and motor scores of the BSID-III and the OX-NDA (Table 3, Pearson’s *r* = 0.50-0.52, *p* < 0.001). The ICCs for the BSID-III and OX-NDA domains showed similarly strong associations for cognition and motor outcomes (ICCs 0.63-0.68, *p* < 0.001), and low associations for language outcomes (ICC 0.30, *p* = 0.04).

The Bland-Altman analysis (Table 4) indicated no, or very low, bias in the subscales, suggesting little to acceptable difference between OX-NDA and BSID-III scores [32]. The Bland-Altman plots (Additional file 4: Fig. S4) and the linear regression analyses (Table 4) of difference scores (BSID-III *minus* OX-NDA) revealed positive associations between the measures.

**Table 4:** Evaluation of agreement between OX-NDA and BSID-III

OX-NDA Domain (*n* = 104)	BSID-III composite scores (*n* = 104)	Correlation analysis		Bland Altman Analysis			Linear regression of difference scores^1^	Comparison of OX-NDA domain scores between children scoring ≥85 and < 85 on the BSID-III		
		Pearson’s r (p)	ICC (95% CI, p)	Bias	Lower limit of agreement	Upper limit of agreement	r	Children scoring < 85 on the BSID-III Mean (SD)	Children scoring ≥85 on the BSID-III Mean (SD)	t (p)
Cognition	Cognition	0.50** (< 0.001)	0.66** (0.50-0.77, < 0.001)	0.10	−67.88	−23.88	0.16**	50.42 (10.66)	65.82 (9.77)	3.08** (< 0.001)
Motor	Motor	0.52** (< 0.001)	0.68** (0.53-0.78, < 0.001)	0.36	−42.33	0.84	0.27**	60.31 (14.28)	80.68 (9.54)	6.09** (< 0.001)
Language	Language	0.18 (0.07)	0.30* (− 0.04 – 0.52, 0.041)	0.08	−77.42	−24.28	0.28**	53.12 (11.26)	61.72 (9.49)	1.54 (0.13)

*OX-NDA* The Oxford Neurodevelopment Assessment; BSID-III: The Bayley Scales of Infant Development III edition; Pearson’s r: Pearson’s correlation coefficients; ICC: interclass correlation coefficients; CI: Confidence intervals; t: independent sample t test

**p < 0.001, *p < 0.05

^1^Difference scores were calculated as BSID-III *minus* OX-NDA scores

### Sensitivity and specificity analysis

Fewer than 10% of the study’s children obtained BSID-III composite scores < 85, no children scored < 70. Children scoring low (< 85) on the BSID-III composites also scored low on the OX-NDA across all domains (Table 4). The sensitivity and specificity of the OX-NDA cognition and motor scores in predicting moderate to severe delay on the BSID-III (composite scores < 85) was strong (Additional file 5: Table S5) at cut-offs of ≤60 (sensitivity 76%, specificity 75%), ≤73 (sensitivity 73%, specificity 80%), and ≤ 71 (sensitivity 70%, specificity 25%), respectively. This was low for the OX-NDA language score at a cut-off of ≤60 (sensitivity 43%, specificity 33%) [34, 35].

### Reliability analysis

The inter-rater and test-retest reliability was high (Cohen’s *k* = 0.80-0.96, 95% CI: 0.78-0.97 and Cohen’s *k* = 0.85-0.94, 95% CI: 0.80-0.95) across all OX-NDA domains (Table 5).

**Table 5:** Inter-rater and test-re test reliability of the OX-NDA

OX-NDA domain	Kappa	95% CIs
Inter-rater reliability		
Cognition	0.87	0.84-0.90
Motor	0.96	0.95-0.97
Language	0.84	0.80-0.88
Positive Behaviour	0.82	0.79-0.86
Negative Behaviour	0.80	0.78-0.85
Test-re test reliability		
Cognition	0.94	0.93-0.95
Motor	0.92	0.89-0.94
Language	0.89	0.85-0.93
Positive Behaviour	0.86	0.83-0.90
Negative Behaviour	0.85	0.80-0.87

*OX-NDA* The Oxford Neurodevelopment Assessment; CI: Confidence intervals

VI. Feasibility Analysis for Use Across Populations and Settings.

The OX-NDA was assessed against three international guidelines for measuring ECD in low- and middle-income settings (Table 6): the World Bank’s Toolkit for Measuring ECD [10]; Fischer et al’s feasibility criteria for use of developmental screening tools at primary healthcare level in low- and middle-income settings [11] and Boggs’ et al’s rating of ECD measurement tools for routine health programme use [9]. The OX-NDA met all but one World Bank and Fischer criteria, and scored 19 (of a maximum total of 24) on Boggs et al’s rating.

**Table 6:** Assessment of the OX-NDA against feasibility criteria for use of an early child developmental assessment in a low- and middle-income setting

Feasibility Criteria	Does OX-NDA fulfil the criteria?	Additional details
World Bank Toolkit for Examining ECD^1^		
Psychometrically adequate, valid and reliable	Yes	ICCs 0·63 and 0·68 (*p* < 0·001) between BSID-III and OX-NDA for cognition, and total score domains; and motor composite; ICC 0.30 (*p* < 0.04) for language composite. Internal consistency satisfactory. Sensitivity in predicting BSID-III composite scores < 85 (moderate delay) was 76, 73, and 43% for the OX-NDA cognition, motor and language domains at cut-off scores of <=60, 73, and 60 respectively. Specificity in predicting BSID-III composite scores < 85 (moderate delay) was 75, 80, and 33% for the OX-NDA cognition, motor, and language domains at cut-off scores of <=60, 73, and 60 respectively. Inter-rater reliability and test-rest reliability was *k* = 0.80-0.96, 95% CI: 0.78-0.97 and *k* = 0.85-0.94, 95% CI: 0.80-0.95 across all domains.
Balanced in terms of number of items at the lower end to avoid children with low scores	Yes	Age range of items extend to 6 months
Enjoyable for children to take (e.g. interactive, colourful materials)	Yes	
Relatively easy to adapt to various cultures	Yes	Adapted via cultural customisation session during training and currently in use in Brazil, India, and Grenada
Easy to use in low-resource settings, e.g. not requiring much material	Yes	Cost of kit GBP 100.0; no fee per use; manuals and forms available upon request, mobile phone/tablet based OX-NDA E-form available.
Not too difficult to obtain or too expensive	Yes	See above
Able to be used in a wide age range	No	Moderately narrow age range (10 to 14 months)
Fischer et al’s feasibility criteria for use of developmental screening tools at primary healthcare level in low-middle income settings^2^		
Results understood by health workers	Yes	Cut-offs for moderate-to-severe delay
Reliable	Yes	High, inter-rater reliability and test-rest reliability of *k* = 0.80-0.96, 95% CI: 0.78-0.97 and *k* = 0.85-0.94, 95% CI: 0.80-0.95 across all subscales.
Valid	Yes	See above
Acceptable to caregivers	Yes	
Provides information that is relevant to primary care providers	Yes	Cut-offs
Information that can be used for referrals of early intervention	Yes	Cut-offs
Information that is useful for anticipatory guidance	Unknown	
Results understood by caregivers	Yes	
Staff members have the expertise to answer questions	Yes	Session on maternal questions and responses included in training package.
Access to application	Yes	Manuals, paper forms and E-form available upon request.
Training involved	Yes	Time taken to train assessors in the OX-NDA: 1 day for ≤3 assessors, 2 days for 3-5 assessors, 3 days for 5-10 assessors
How long it takes to administer the tool	Yes	15-25 minutes
Cover multiple areas of child development	Yes	Cognition, language, motor skills, and behaviour (positive and negative)
Cost of the tool	Yes	Cost of kit GBP100.0; no fee per use; mobile phone/tablet based OX-NDA E-form available.
Minimal adaptation needed	Yes	Sessions on cultural customisation and translation included in training
Educational level of staff members	Yes	Primary education; non-specialist personnel
How many staff members to administer the tool	Yes	1
Local norms available	Yes	Cut-offs based on Brazilian sample. Research to develop international norms on-going.
Space	Yes	Minimal space for storage of kit and forms. Mobile phone/tablet based OX-NDA E-form available. Home-based assessments possible.
Boggs et al’s rating of early child development outcome measurement tools for routine health programme use^3^		
Validity	Rating: 2	Validity somewhat below widely accepted threshold (0.5 to 0.7) against another performance-based tool e.g. BSID-III
Reliability	Rating: 3	High, inter-rater reliability and test-rest reliability of k = 0.80-0.96, 95% CI: 0.78-0.97 and *k* = 0.85-0.94, 95% CI: 0.80-0.95 across all subscales.
Cultural Adaptability	Rating: 3	Easy modification of items, materials and procedures
Accessibility	Rating: 2	Tool administration, scoring and interpretation, all available online, but some intellectual property or other restrictions. Minimal cost to tool <US$ 10 per child App (mobile phone/tablet based OX-NDA E-form) available
Training	Rating: 2	Moderate (> 1 hour to < 1 day), requires standardization and training on direct assessment of children’s abilities, no certification requirement.
Administration time	Rating: 2	< 15 to 20 minutes, minimum to moderate scoring.
Geographical uptake	Rating: 3	Used in at least three continents (Asia, Europe, South America)
Clinical relevance and utility	Rating: 2	Sensitivity in predicting BSID-III composite scores < 85 (moderate delay) was 76, 73, and 43% for the OX-NDA cognition, motor and language domains at cut-off scores of <=60, 73 and 60 respectively. Specificity in predicting BSID-III composite scores < 85 (moderate delay) was 75, 80, and 33% for the OX-NDA cognition, motor and language domains at cut-off scores of <=60, 73, and 60 respectively. Further research to develop international norms, and contextually appropriate referral pathways underway.

*ECD* Early child development

*OX-NDA* The Oxford Neurodevelopment Assessment

*BSID-III* The Bayley Scales of Infant Development III edition

*ICC* interclass correlation coefficients

*CI* Confidence intervals

*k* Cohen’s kappa coefficient

^1^Fernald LCH, Kariger P, Engle P, et al. Examining Early Child Development in Low-Income Countries: A Toolkit for the Assessment of Children in the First 5 Years of Life. Washington DC: The World Bank, 2009

^2^Fischer VJ, Morris J, Martines J. Developmental screening tools: feasibility of use at primary healthcare level in low-and middle-income settings. Journal of health, population, and nutrition 2014;32(2):314

^3^Boggs D, Milner KM, Chandna J, et al. Rating early child development outcome measurement tools for routine health programme use. Archives of disease in childhood 2019;104 (Suppl 1):S22-S33

## Discussion

The OX-NDA is a multi-dimensional, mixed methodology instrument for measuring cognitive, motor and behaviour outcomes in children aged 10 to 14 months: its psychometric validity was low for the language domain. The rationale for its construction was to provide a comprehensive neurodevelopmental assessment that can be administered reliably and rapidly to young children by non-specialists in low-resource settings at relatively low costs. The preliminary evidence obtained from this study supports the OX-NDA’s use as a valid and reliable measure of early cognitive, motor and behaviour outcomes in Brazilian infants.

Across cognitive and motor domains, the OX-NDA demonstrated good concurrent validity with the BSID-III, satisfactory psychometric properties and high levels of inter-rater and test-retest reliability. Importantly, its sensitivity and specificity in predicting moderate-to-severe cognitive and motor delay in 1-year-olds was satisfactory. The OX-NDA’s concurrent validity with the BSID-III for the language domain was low. It is possible that this may reflect the observation that language, as a construct, is highly influenced by culture and it was, as such, not possible for us to ascertain from our dataset whether it is the OX-NDA or the BSID-III that have limited function in this context.

Across domains, the OX-NDA’s reliability, when administered by non-specialists, was high and its administration time was shorter and cost per child was lower than comparable measures. Previous studies have shown that ECD assessments for older ages and constructed using similar approaches, such as the INTER-NDA [22] and TADI [20], have yielded similar results.

Our study was limited in that all children were Brazilian, recruited from the city of Pelotas, and that the comparison of the OX-NDA’s behaviour domain with the BSID-III was not ascertained. Its agreement with the BSID-III language composite was limited and further work is needed to ascertain why this was the case and how its performance in the language domain may be improved. Moreover, its ability to predict severe delay could not be determined as no children in our study scored < 70 on the BSID-III composite scores. Additionally, the OX-NDA’s age range is narrow (10 to 14 months). Finally, item selection was guided by the expert panel, initial piloting and assessment of internal consistency: we did not conduct a factorial analysis for item selection which limited our ability to scrutinise relations between observed and latent variables.

Nevertheless, the study’s strengths lie in the detailed validation of the OX-NDA, using multiple statistical techniques, in a population-based sample from a low-and-middle-income setting against the BSID-III [25], a well-established measure of ECD, considered, by some, to be a gold-standard ECD assessment. Our study design controlled for child fatigue and contamination of results during sequential developmental testing sessions.

The OX-NDA offers several practical and conceptual advantages over other infant developmental assessments (Table 6, Additional file 1: Table S1). First, it reliably measures multiple domains of infant development (cognition, motor, and behaviour). For each domain, outcomes are reported on a 5-point scale characterising the child’s performance across a spectrum and offering a level of granularity beyond that provided by many infant development batteries. Second, it was designed to be free from cultural biases and is based upon objective reporting (rather than subjective judgement) of the child’s performance. Its rigorous standardization protocol ensures children are assessed uniformly and reliably. Moreover, it can be administered reliably by non-specialist assessors in a short assessment time and at relatively low costs. Third, because it is a mixed-methodology assessment, it applies the advantages of direct assessment as well as caregiver- and observer-reports. It assesses cognitive processing by presenting children with new tasks and captures previously demonstrated abilities while being minimally affected by reporter and recall biases.

Nevertheless, although the OX-NDA fulfilled most requirements for a population-level ECD measure when assessed against the World Bank’s [10], Fischer et al’s [11] and Boggs’ et al’s criteria [9] (Table 6); the findings of this study may not be generalizable to culturally and linguistically disparate LMICs and further research is needed to evaluate its adaptability and applicability in screening for infant development delay in populations beyond Brazil. Additionally, further work is needed to examine (and improve) its performance in the language domain. Currently, such studies are on-going in the West Indies, Indonesia, India, and Senegal.

Identifying children at risk of delay during early childhood and comparing outcomes across populations are essential prerequisites for achieving indicator 4.2.1 of the Sustainable Development Goals. The OX-NDA measure presented here contributes an important component to the care of young children: a unique, standardized developmental tool that can be applied by non-specialists in low-resource settings to measure neurodevelopmental outcomes in infants reliably, rapidly and at relatively low costs and to identify those children at risk of delays.

## Conclusions

The evidence presented here shows the OX-NDA to be a psychometrically sound measurement tool for the assessment of cognitive, motor and behaviour outcomes in Brazilian infants. Its mixed-methodology, multidimensional approach; relatively low costs; and reliability when administered by non-specialists make it an attractive candidate ECD measure for research and clinical efforts aimed at identifying infants at risk of delay in low-resource settings. Its ability to identify language delay in Brazilian infants was low and further work is needed to examine (and improve) its performance in the language domain. Research efforts, focusing on its adaptation and application in LMIC settings beyond Brazil, are on-going.

## Supplementary Information

**Additional file 1: Table S1.**

**Additional file 2: Table S2.**

**Additional file 3: Table S3.**

**Additional file 4: Fig. S4.**

**Additional file 5: Table S5.**

## Data Availability

The data that support the findings of this study are available from the DOVE research centre (contact@doveresearch.org) but restrictions apply to the availability of these data, which were used under license for the current study, and so are not publicly available. Data are however available from the authors/corresponding author upon reasonable request and with permission of the DOVE research centre (contact@doveresearch.org).
