# Supplementary material for: Construction and validation of the Oxford Neurodevelopment Assessment (OX-NDA) in 1-year-old Brazilian children
Source: BMC Pediatr. 2022 Dec 23;22:733. doi: 10.1186/s12887-022-03794-1 (PMC9783969; doi:10.1186/s12887-022-03794-1)
Supplement: Supplementary file 1 — Additional file 1: Table S1. [file 12887_2022_3794_MOESM1_ESM.pdf]

ADDITIONAL FILE 1

Table S1 Characteristics of International Developmental Assessments for Infants Aged 12 Months.

Tool	Subscales measured	Characteristics of normative sample	Cut-off score for neurodevelopmental delay	Assessment type	Assessors' qualifications	Administration time	Psychometric information
The Bayley Scales of Infant Development – III edition (BSID III) [a]	Cognitive, expressive and receptive communication, fine and gross motor, adaptive behaviour, social emotional.	Country: USA<br>Year: January and October 2004<br>Sample Size: 12-month normative sample for cognitive, language and motor scales: 100 children (totally 1700 children aged 16 days through 43 months 15 days divided into 17 age groups of 100 children each). Normative sample for the social-emotional scale was based on 456 children and the adaptive behaviour scale was based on 1,350 children.<br>Sample characteristics: Sample selected to match the 2000 United States census.	Multiple criteria: **25% delay** in functioning when compared to same age peers; based on SD (**< -1 SD** i.e. cut-off thresholds of 85 for moderate impairment; **< -2 SD** i.e. cut-off thresholds of 70 for severe impairment) or **performance of a certain number of months below** the child's chronological age[1].	Structured direct administration	Child health specialists and healthcare professionals with training & credentials	60—90 minutes	Test-re test and inter-rater reliability: acceptable to high<br>Internal consistency: high<br>Predictive validity: high
The Malawi Developmental Assessment Tool (MDAT)[b]	Gross motor, fine motor, language and social.	Country: Malawi<br>Year: June 2006 to July 2007<br>Sample size: 1426 normal healthy children aged 0 to 6 years<br>Sample characteristics: Those born preterm at <32 weeks' gestation, or with significant malnutrition using WHO criteria, medical problems or significant neurodisability were excluded.	**<25%** (upper limit of lowest quartile); Plot children on MDAT normal reference ranges, corresponding to 25%, 50% and 75% of children passing each item.	Direct administration	Trained professionals and para-professionals	35 minutes	Test-re test and inter-rater reliability: Good, k>0.4<br>Sensitivity: 97%<br>Specificity: 82%
The Griffiths Mental Development Scales[c]	Locomotor, expressive and receptive language, personal-social, hand and eye coordination, performance, practical reasoning.	Country: UK and Ireland<br>Year: 1960s, revised in 2015<br>Sample size: 1026 children<br>Sample characteristics: National representative sample of children in UK; stratified according to geographical region and proportionate to the 1997 ONS population ratios[1].	Centiles and z -scores; **z score < -2 or SD< -2** indicates significant developmental delay on that subscale.	Structured direct administration	Healthcare professionals who have completed a 5 day accredited training course	60 minutes	The publishers state that, except for Scale E (performance), in children aged less than 48 months the coefficients 'all comfortably exceed the minimum acceptable value of 0.70'
Pre-school version of	Problem and syndrome scales	Country: USA<br>Year: 1979, 2000	**>93rd centile** is abnormal (norms vary according to	Caregiver report	Parents or teachers,	10 to 20 minutes	Test-re test and inter-rater reliability:

Child Behavior Checklist (CBCL)[d]	for emotionally reactive; anxious/depressed; somatic complaints; withdrawn; sleep problems; attention problems; aggressive behaviour.	Sample size: Originally normed on 1728 US children.<br>Sample characteristics: -<br><br>Note: Multicultural norms available.	societies, and map onto the Diagnostic and Statistical Manual for Mental Disorders).<br><br>Any score that falls below the 93rd centile is considered normal, scores between the 93-97th centile are borderline clinical, and any score above the 97th centile is considered to be in the clinical range.		scoring restricted to qualified professionals		kappa=0.8; 0.61<br>Internal consistency: good<br>Predictive validity: high<br>Sensitivity: 84%
The Rapid Neurodevelopmental Assessment (RNDA)[e]	Primitive reflexes, gross motor, fine motor, vision, hearing, speech, cognition, behaviour, and seizures.	Country: Bangladesh<br>Year: 2010<br>Sample size: 81 children aged ≥3 to 24 months in urban (n = 47) and rural (n = 34) community-based populations<br>Sample characteristics: 15% did not 'look' properly nourished, parental concerns regarding child development were expressed in 8% and for 50% at least one parent was illiterate.	**< -2 SD**: threshold for severe impairment;<br>**< -1 SD:** threshold for mild impairment.<br>If low scores in >1 domain; the child is classified as having 'any' neurodevelopmental impairment.	Direct administration	Trained professionals and para-professionals who have completed an accredited training course	30 minutes	Inter-rater reliability: Good to excellent k=0.63-1.00<br>Validity: The authors state " good concurrent validity (ie, significantly lower mean Mental Development Index and Psychomotor Development Index scores) for children with >1 neurodevelopmental impairment and for children with impairments in most functional domains, compared with children with no impairments".
The Ages and Stages Questionnaire III edition (ASQ III)[f]	Communication, gross motor, fine motor, problem solving and personal-social.	Country: USA<br>Year: June 2008<br>Sample Size: 15,138 children.<br>Sample characteristics: 76% of the sample had one or no known risk factor, 19% had 2 risk factors and 4% 3 or more risk factors. Risk factors were defined as extreme poverty, maternal age ≤19 years, maternal education <12th grade; involvement of child protective services with the family for abuse and/or neglect; medical risk, including prematurity; and infant's birth weight	**< -2SD**	Caregiver report	Parents, caregivers, home visitors; requires a 6th grade reading level.	15-20 minutes	Test-re test and inter-rater reliability: high<br>Internal consistency: moderate<br>Predictive validity: moderate<br>Sensitivity: 38% to 90%<br>Specificity: 81 to 90%

		less than 3 pounds, 5 ounces.					
The Parents' Evaluation of Developmenta l Status (PEDS)[g]	General development.	Country: USA & Canada<br>Year: 2012<br>Sample size: 47531 families, of which 13,523 were aged 0-11 months<br>Sample characteristics: Families from varying backgrounds, including SES and ethnicity.	Table for using scores to identify parental difficulties, non-significant concerns, one significant concern or two or more significant concerns by shading boxes based on scores. These are then used to select associated algorithms for further screening and/or referral.	Caregiver report	Parents	2-10 minutes	Reliability: 94% acceptable<br>Sensitivity: 74% to 80%<br>Specificity: 70 to 80%
Caregiver-reported Early Developmenta l Instruments (CREDI)[h]	Long Form: Motor, cognitive, language, social-emotional and overall.<br><br>Short form: Overall development.	Countries: Brazil, Cambodia, Chile, Colombia, Ghana, Guatemala, India, Nepal, Philippines and USA<br>Year: 2017-2018<br>Sample size: 7807 children aged 0-35 months<br>Sample characteristics: Children with an "ideal home environment" defined through maternal educational attainment<br>(college or higher), and the number of activities done by adults with the child in the last 3 days (at least 4 out of the 6 MICS home stimulation activities); authors acknowledge that "although the data were representative for local populations in Brazil, Ghana, Tanzania, and Zambia, the overall sample is not representative of any country or a global population of children".	Raw scaled scores, **norm referenced standardised scores**, and **z scores**.	Caregiver report	Parents	Short Form <5 minutes, Long Form 15 minutes	Test-re test reliability: 0.62<br>Internal consistency: 0.84 for 12-17 months<br>Correlation with other measures (ASQ, BSID, INTER-NDA, McAruthr Bates Inventory, PRIDI): r=0.2-0.4
Denver Development Screening Test II (DDST II)[i]	Personal social, fine motor adaptive, language and gross motor.	Country: USA<br>Year: 1980s and 1990<br>Sample size: 2096 children<br>Sample characteristics: Children from Colorado; based on 1988, 1989 and then the 1990 US census population.	**Centile ranks** (25th, 50th, 75th, and 90th) are displayed as bar graphs and reflect the ages at which 25%, 50%, 75% and 90% of typically developing children in the standardisation sample completed the task.<br>Overall categories: Normal, and suspect.	Direct administration	Trained professionals and paraprofession als	15-20 minutes	Test-re test reliability: 0.9<br>Inter-rater reliability: 0.99<br>No validity study available.
Guide for Monitoring Child Development (GMCD)[j]	Parental concerns, expressive language and communication,	Country: Turkey<br>Year: 1980s and 1990<br>Sample size: 4949 children up to 42 months of age from primary healthcare centres in Argentina, India, South Africa and Turkey	Paper 1: **<10th centile** for any domain; if a child did not demonstrate ≥1 of the age-appropriate milestones, the GMCD interpretation was	Open ended caregiver interview	Trained providers completing the GMCD Provider	7 ± 2.3 minutes	Inter-rater reliability: 0.83-0.88<br>Internal consistency: 0.28-0.91<br>Sensitivity: 0.71-0.94

	receptive language, relationship (social-emotional), play (social-emotional and cognitive) and self-help.	Sample characteristics: Children were born healthy singletons with birth weight ≥2500 g and gestational age ≥37 weeks, with postnatal growth was between the 5th and 95th centiles since birth; free from chronic health and developmental problems or anemia (Hemoglobin <10.5 g/dL) at recruitment	classified as "requires follow-up evaluation with or without intervention."<br>Paper 2**: thresholds based on BSID-III cut-offs**		Training Program		Specificity: 0.69-0.82
Development Assessment Scale for Indian Infants (DASII)[k]	Mental and Motor Development	Country: India<br>Year: 1996<br>Sample size: 513 aged 1 to 30 months<br>Sample characteristics: Certified as normal and healthy by a pediatrician	Developmental quotients calculated from motor age (MoA) and mental age (MeA), percentile ranks for developmental quotients	Structured direct administration	Child health specialists and healthcare professionals with training & credentials	30-40 minutes	Inter correlation between motor and mental performance: 0.24 to 0.62<br><br>Median reliability Index: 0.88 (Motor), 0.91 (Mental)
Test de Aprendizaje y Desarollo Infantil (TADI)[l]	Cognition, motor, language, and social-emotional.	Country: Ecuador<br>Year: 2016-2020<br>Sample size: 411 children aged 12 to 36 months<br>Sample characteristics: Convenience sample	Ecuador norm based **z score**	Direct administration, caregiver reported	Trained specialists	20-30 minutes	Test re test reliability: Good, z score 0.19<br>Internal consistency: 0.97<br>Validity: Concordance of 90% with expert judgment of the child's abilities
Indicators of Infant and Young Child Development (IYCD)[m]	Fine motor, gross motor, receptive language, expressive language, and socio-emotional.	Country: Meta-data analysis of datasets from Costa Rica, Nicaragua, Paraguay, Peru, Bangladesh, India, Indonesia, Kenya, Malawi, and Tanzania.<br>Year: 2014-2018<br>Sample size: 14 cross sectional datasets representing data from 7 tools from 21 083 children from 10 low/middle- income countries<br>Sample characteristics: authors were in broad agreement as to the important domains of development, which aligned with the review findings; sampled children came from diverse socioeconomic backgrounds with the highest proportions classified as 'normal' by height for age z-score (HAZ) and weight for age z-score (WAZ); and most mothers had been educated to at least primary level.	**Item response theory curves** from fitted models depicting the ages at which 10% (lower limit), 50% (diamond) and 90% (upper limit) of children pass the item for each item group.	Caregiver report	Not specified	Not reported	Feasibility study ongoing, validity study imminent.
Battelle's	Cognitive,	Country: USA	**Basal and ceiling level** for	Structured	Child health	90	Test-re test and inter-

Development Inventory (BDI)[n]	adaptive (self-help), motor, communication, and personal-social development	Year: c1988<br>Sample size: 50 children aged 12-17 months<br>Sample characteristics: 75% urban, 25% rural, 50% girls, 50% boys, 84% Caucasian, 16% ethnic minorities.	domain scores, **developmental quotients**, expressed as age equivalents (in months)	administration, observation, and interviews with parents or other sources	specialists and healthcare professionals with training & credentials	minutes	rater reliability: 0.99<br>Internal consistency: >0.85<br>Construct validity: High<br>Concurrent validity: r=0.78-0.93 with the Vineland Social Maturity Scale and the Developmental Activities Screening Inventory; r=0.40-0.61 with the the Stanford-Binet Intelligence Test
Mullen Scales of Early Learning (MSEL)[o]	Gross Motor and four "cognitive scales": Visual Reception, Fine Motor, Expressive Language, and Receptive Language	Country: USA<br>Year: 1981-1989<br>Sample size: 1849 children aged 0-68 months<br>Sample characteristics: not mentioned	Raw domain scores compared against **age equivalents**. "Cognitive" scores summarized into an **Early Learning Composite**. Scores mapped to obtain **percentile ranks** and age equivalent scores.	Structured direct administration	Child health specialists and healthcare professionals with training & credentials	30-45 minutes	Limited evidence for concurrent, content and construct validity.
Comprehensive Developmental Inventory for Infants and Toddlers (CDIIT)[p]	Cognition, motor skills, language, self help and social development	Country: Taiwan<br>Year: c1998<br>Sample size: 3703 aged 3 to 72 months<br>Sample characteristics: The authors state that the sample was "randomly selected according to age, sex, urbanization, and geographic regions in Taiwan"	Items are scored 0 or 1 (fail or pass), developmental quotients (DQs) calculated based on Taiwanese norms. **DQ ≥ 85 normal, 70-84 borderline, <70 indicates delay**.	Structured direct administration	Trained specialists	Not reported	Test-re test and inter-rater reliability: ICC 0.76-1.00<br>Internal consistency: 0.75-0.99<br>Sensitivity: 87%<br>Specificity: 97%

ICCs = interclass correlations.
[a]BSID-III: Bayley Scales of Infant and Toddler Development, Third Edition (Bayley-III); ©Pearson. Available from: http://www.pearsonclinical.co.uk
[b]MDAT: The Malawi Developmental Assessment Tool; Gladstone M, Lancaster GA, Umar E, Nyirenda M, Kayira E, van den Broek NR, Smyth RL. The Malawi Developmental Assessment Tool (MDAT): the creation, validation, and reliability of a tool to assess child development in rural African settings. *PLoS medicine* 2010; **7:** e1000273.
[c]Griffiths: Griffiths Mental Development Scales; Luiz D, Barnard A, Knoesen N, Kotras N, Horrocks S, McAlinden P, Challis D, O'Connell R. Griffiths Mental Development Scales—Extended Revised: Two to Eight Years: Administration Manual. Hogrefe, Oxford, UK 2006.
[d]CBCL: Preschool Child Behavior Checklist; Achenbach TM. Manual for the Child Behavior Checklist/4-18 and 1991 profile. University of Vermont, Department of Psychiatry 1991.
[e]RNDA: Rapid Neurodevelopmental Assessment; Khan NZ, Muslima H, Begum D, Shilpi AB, Akhter S, Bilkis K, Begum N, Parveen M, Ferdous S, Morshed R, Batra M. Validation of rapid neurodevelopmental assessment instrument for under-two-year-old children in Bangladesh. Pediatrics. 2010 Apr 1;125(4):e755-62.
[f]ASQ-III: Ages & Stages Questionnaires®, Third Edition**;** https://agesandstages.com/products-pricing/asq3/

[g]PEDS: Parents' Evaluation of Developmental Status. Glascoe FP. Collaborating with parents: Using Parents' Evaluation of Developmental Status to detect and address developmental and behavioral problems. Ellsworth & Vandermeer Press; 1998. Available at: http://forepath.org/.
[h]CREDI: Caregiver Reported Early Developmental Instruments; McCoy DC, Waldman M, Team CF, Fink G. Measuring early childhood development at a global scale: evidence from the Caregiver-Reported early development instruments. Early childhood research quarterly. 2018 Oct 1;45:58-68. https://sites.sph.harvard.edu/credi/
[i]DDST-II: The Denver Developmental Screening Test IInd edition; Frankenburg WK, Dodds J, Archer P, Shapiro H, Bresnick B. The Denver II: a major revision and restandardization of the Denver Developmental Screening Test. *Pediatrics* 1992; 89**:** 91-7.
[j]GMCD: A Guide for Monitoring Child Development; Ozturk Ertem, I., Krishnamurthy, V., Mulaudzi, M.C., Sguassero, Y., Bilik, B., Srinivasan, R., Balta, H., Gulumser, O., Gan, G., Calvocoressi, L., Johnson, B., Shabanova, V. and Forsyth, B.W. (2019), Validation of the International Guide for Monitoring Child Development demonstrates good sensitivity and specificity in four diverse countries. Acta Paediatr, 108: 1074-1086.
[k]DASII: Developmental Assessment Scales for Indian Infants; Phatak P. Mental and Motor Growth of Indian Babies (1 Month-30 Months).(Longitudinal Growth of Indian Children). Final Report. https://www. manashakti.org/tests/developmental-assessment-scales-indian-infants
[l]TADI: Test de Aprendizaje y Desarrollo Infantil; López Vanegas, N. y Peñafiel Aguirre, T. (2020). Adaptación y validación del test de Aprendizaje y desarrollo infantil "TADI" en el GAD de Calderón. Trabajo de titulación previo a la obtención del Título de Psicólogo Infantil y Psicorehabilitación. Carrera de Psicología Infantil y Psicorehabilitación. Quito: UCE. 190 p. http://www.dspace.uce.edu.ec/bitstream/25000/20829/1/T-UCE-0007-CPS-245.pdf
[m]IYCD: WHO Indicators of Infant and Young Child Development; Lancaster GA, McCray G, Kariger P, *et al*. Creation of the WHO Indicators of Infant and Young Child Development (IYCD): metadata synthesis across 10 countries. *BMJ Glob* 72 *Health* 2018;3:e000747.
[n]BDI-2: Battelle's Development Inventory 2; Newborg, J. , Stock, J.R. , Wnek, L. , Guidabaldi, J. , & Svinicki, J. (1984). Battelle Developmental Inventory Screening Test. Allen Texas: DLM-Teaching Resources. Abbey Tyner Berls, Irene R McEwen, Battelle Developmental Inventory, *Physical Therapy*, Volume 79, Issue 8, 1 August 1999, Pages 776–783.
[o]MSEL: Mullen's Scales of Early Learning; Mullen, E. *Mullen Scales of Early Learning*. Circle Pines, NM: American Guidance Service 1995.
[p]CDIIT: Comprehensive Developmental Inventory for Infants and Toddlers; Hwang AW, Weng LJ, Liao HF. Construct validity of the comprehensive developmental inventory for infants and toddlers. Pediatrics international. 2010 Aug;52(4):598-606.
