# Supplementary material for: Construction and validation of the Oxford Neurodevelopment Assessment (OX-NDA) in 1-year-old Brazilian children
Source: BMC Pediatr. 2022 Dec 23;22:733. doi: 10.1186/s12887-022-03794-1 (PMC9783969; doi:10.1186/s12887-022-03794-1)
Supplement: Supplementary file 2 — Additional file 2: Table S2. [file 12887_2022_3794_MOESM2_ESM.pdf]

ADDITIONAL FILE 2

Table S2 Mapping analysis of International Developmental Assessments for Infants Aged 12 Months.

Devel op-menta l Domai ns	ECD Conceptual constructs	ECD Conceptual elements contained in 10-14 month battery															
		BSID-III[1]	MDAT[2]	Griff-iths[3]	CBCL[4]	RNDA[5]	ASQ-III[6]	PEDS[7]	CRE-DI[8]	DDST-II[9]	GMCD[10]	DAS II[11]	TADI[12]	IYCD[13]	BDI-2[14,a]	MSEL[15,a]	CDIIT[16]
**Conceptual constructs according to OX-NDA working group's neurodevelopmental matrix (Age Band: 10-14 months)**																	
Cognit-ive	Comprehension of simple instructions																
	Constructional skills																
	Comprehends concept of 'one'																
	Object permanence																
	Basic problem solving skills																
	Object matching/sorting																
	Pretend play																
Motor	Pincer grasp and grasp release																
	Transfer of objects between hands																
	Walks with support/alone																
	Stands alone																
	Motor coordination																
Lang-uage	2-4 syllable babble																
	1 meaningful word																

	2 words together																
	Preverbal gesture																
	Comprehends own name																
	Comprehends concept of 'no'																
Execu-tive Funct-ion	Multi-step problem solving																
	Demonstrates means to an end																
	Empathy																
Behav-iour	Positive behavior attributes																
	Negative behavior attributes																
	Emotional lability																
	Emotional regulation																
	Curiosity																

ECD Conceptual elements as per WHO IYCD indicators (Age Band: 12.1-18.0 months)

		**BSID-III**[1]	**MDAT**[2]	**Griff-iths**[3]	**CBCL**[4]	**RNDA**[5]	**ASQ-III**[6]	**PEDS**[7]	**CRE-DI**[8]	**DDST-II**[9]	**GMCD**[10]	**DAS II**[11]	**TADI**[12]	**IYCD**[13]	**BDI-2**[14,a]	**MSEL**[15,a]	**CDIIT**[16]
Cognitive	Object sorting																
Motor	Matured motor coordination																
Language	Use of at least two to four words																
Social-emotional	Stranger anxiety																
	Self control/delay in gratification																
	Emergence of compliance																

Legend:

(green)	Specific construct clearly present
(blue)	Related construct present
(orange)	General overview of parental concern only
(yellow)	Construct not present
(grey)	Unable to assess as information not freely available

[1]BSID-III: Bayley Scales of Infant and Toddler Development, Third Edition (Bayley-III); ©Pearson. Available from: http://www.pearsonclinical.co.uk
[2]MDAT: The Malawi Developmental Assessment Tool; Gladstone M, Lancaster GA, Umar E, Nyirenda M, Kayira E, van den Broek NR, Smyth RL. The Malawi Developmental Assessment Tool (MDAT): the creation, validation, and reliability of a tool to assess child development in rural African settings. *PLoS medicine* 2010; **7:** e1000273.
[3]Griffiths: Griffiths Mental Development Scales; Luiz D, Barnard A, Knoesen N, Kotras N, Horrocks S, McAlinden P, Challis D, O'Connell R. Griffiths Mental Development Scales—Extended Revised: Two to Eight Years: Administration Manual. Hogrefe, Oxford, UK 2006.
[4]CBCL: Preschool Child Behavior Checklist; Achenbach TM. Manual for the Child Behavior Checklist/4-18 and 1991 profile. University of Vermont, Department of Psychiatry 1991.
[5]RNDA: Rapid Neurodevelopmental Assessment; Khan NZ, Muslima H, Begum D, Shilpi AB, Akhter S, Bilkis K, Begum N, Parveen M, Ferdous S, Morshed R, Batra M. Validation of rapid neurodevelopmental assessment instrument for under-two-year-old children in Bangladesh. Pediatrics. 2010 Apr 1;125(4):e755-62.
[6]ASQ-III: Ages & Stages Questionnaires®, Third Edition**;** https://agesandstages.com/products-pricing/asq3/
[7]PEDS: Parents' Evaluation of Developmental Status. Glascoe FP. Collaborating with parents: Using Parents' Evaluation of Developmental Status to detect and address developmental and behavioral problems. Ellsworth & Vandermeer Press; 1998. Available at: http://forepath.org/.
[8]CREDI: Caregiver Reported Early Developmental Instruments; McCoy DC, Waldman M, Team CF, Fink G. Measuring early childhood development at a global scale: evidence from the Caregiver-Reported early development instruments. Early childhood research quarterly. 2018 Oct 1;45:58-68. https://sites.sph.harvard.edu/credi/
[9]DDST-II: The Denver Developmental Screening Test IInd edition; Frankenburg WK, Dodds J, Archer P, Shapiro H, Bresnick B. The Denver II: a major revision and restandardization of the Denver Developmental Screening Test. *Pediatrics* 1992; 89**:** 91-7.
[10]GMCD: A Guide for Monitoring Child Development; Ertem IO, Dogan DG, Gok CG, Kizilates SU, Caliskan A, Atay G, Vatandas N, Karaaslan T, Baskan SG, Cicchetti DV. A guide for monitoring child development in low-and middle-income countries. *Pediatrics* 2008; 121**:** e581-9.
[11]DASII: Developmental Assessment Scales for Indian Infants; Phatak P. Mental and Motor Growth of Indian Babies (1 Month-30 Months).(Longitudinal Growth of Indian Children). Final Report. https://www. manashakti.org/tests/developmental-assessment-scales-indian-infants
[12]TADI: Test de Aprendizaje y Desarrollo Infantil; López Vanegas, N. y Peñafiel Aguirre, T. (2020). Adaptación y validación del test de Aprendizaje y desarrollo infantil "TADI" en el GAD de Calderón. Trabajo de titulación previo a la obtención del Título de Psicólogo Infantil y Psicorehabilitación. Carrera de Psicología Infantil y Psicorehabilitación. Quito: UCE. 190 p. http://www.dspace.uce.edu.ec/bitstream/25000/20829/1/T-UCE-0007-CPS-245.pdf
[13]IYCD: WHO Indicators of Infant and Young Child Development; Lancaster GA, McCray G, Kariger P, *et al*. Creation of the WHO Indicators of Infant and Young Child Development (IYCD): metadata synthesis across 10 countries. *BMJ Glob* 72 *Health* 2018;3:e000747.
[14]BDI-2: Battelle's Development Inventory 2; Newborg, J., Stock, J.R., Wnek, L., Guidabaldi, J., & Svinicki, J. (1984). Battelle Developmental Inventory Screening Test. Allen Texas: DLM-Teaching Resources.
[15]MSEL: Mullen's Scales of Early Learning; Mullen, E. *Mullen Scales of Early Learning*. Circle Pines, NM: American Guidance Service 1995.
[16]CDIIT: Comprehensive Developmental Inventory for Infants and Toddlers; Hwang AW, Weng LJ, Liao HF. Construct validity of the comprehensive developmental inventory for infants and toddlers. Pediatrics international. 2010 Aug; 52(4): 598-606.
[a] Instrument's commercialization partner contacted for information about the tool, response not obtained.
