# Supplementary material for: Construction and validation of the Oxford Neurodevelopment Assessment (OX-NDA) in 1-year-old Brazilian children
Source: BMC Pediatr. 2022 Dec 23;22:733. doi: 10.1186/s12887-022-03794-1 (PMC9783969; doi:10.1186/s12887-022-03794-1)
Supplement: Supplementary file 3 — Additional file 3: Table S3. [file 12887_2022_3794_MOESM3_ESM.pdf]

ADDITIONAL FILE 3

Table S3 OX-NDA Items and Scoring Options.

No.	Item	Options				
1	Builds a tower of 3 cubes<br>*(Trials = 5, demonstration = 5)*	3 cubes in ≤3 trials	3 cubes in 4-5 trials	2 cubes in 5 trials	No attempt	Unable to assess
2	Takes 3 cubes out of cup (Examiner says "Look, I am putting the cubes into the cup – one, two and three. Can you take them out?")<br>*(Trials =3, demonstration = 0 but visibly place cubes inside cup before asking child to take them out counting as you go)*	Takes all 3 cubes out of the cup in ≤3 trials	Takes 2 cubes out of cup	Takes 1 cube out of cup	No attempt	Unable to assess
3	Puts 3 cubes back in cup (Examiner says 'Can you put the cubes back in the cup?")<br>*(Trials = 3, demonstration = 0)*	Puts all 3 cubes inside cup in ≤3 trials	Puts 2 cubes inside cup in ≤3 trials	Puts 1 cube inside cup in ≤3 trials	No attempt	Unable to assess
4	Hands the examiner one cube when asked to do so (Examiner says 'Please can you give me 1 cube?")<br>*(Trials = 1, demonstration = 0)*	Precise release of one cube into examiner's hand	Clumsy release, cube falls out of examiner's or child's hand, child gives examiner more than one cube	Attempts but unsuccess-ful release with one or more hand	No attempt	Unable to assess
5	Squeeze toy to make sound<br>*(trials = 1, demonstration = 2 squeezes followed by 2 squeezes)*	Squeezes toy to make sound	Squeezes toy but not hard enough to make sound	Picks up toy and plays with it but does not squeeze it	No attempt	Unable to assess
6	Drop squeeze toy on floor (Examiner says "Uh oh, where's ducky	Child looks	Child looks	Child	Child does	Unable to

	gone?")<br>*(trials = 5 following verbal prompts, demonstration = 0)*	towards object or tries to retrieves it spontaneously	towards object or tries to retrieve it in ≤3 prompts	starts looking for fallen toy on repeated prompting (4-5 prompts)	not look for toy even on repeated prompting	assess
7	Finds squeeze toy under cup (Examiner hides toy under cup and says 'Where's ducky gone now?")<br>*(trials = 1, demonstration = 0; verbal prompts = maximum 3)*	Child retrieves toy spontaneously by inverting cup	Child pushes cup or points to cup but does not retrieve toy	Child looks at cup but does not touch it	No attempt	Unable to assess
8	Ask child to give squeeze toy to mummy<br>*(trials = 5, demonstration = 0)*	Child gives toy to mother in in ≤3 trials	Child gives toy to mother with repeated prompting i.e. 4-5 trials	Child does not give toy to mother or gives it and takes it back	No attempt	Unable to assess
9	Identifies spoon out of 5 objects. Examiner says, "Which one is the spoon?"<br>*(trials = 5, demonstration = 0)*	Child correctly identifies spoon in ≤3 trials	Child identifies spoon with repeated prompting i.e. 4-5 trials	Child identifies an object but it is not the spoon	No attempt	Unable to asses
10	Identifies sock and toothbrush out of 5 objects. Examiner says, "Show me the sock and the toothbrush".<br>*(trials = 3, demonstration = 0)*	Child correctly identifies sock and toothbrush correctly	Child identifies one object correctly	Child attempts to identify objects but none are correct	No attempt	Unable to asses

11	Pretends to drink from a toy tea cup when placed in front of him/her<br>*(Trials = 2, demonstration = 1 if not spontaneous on first attempt)*	Spontaneously	After 1 demonstration	Partial attempt after 1 demonstr ation	No attempt	Unable to assess
12	Lifts empty cup by handle<br>*(if not concurrently observed with item above; demonstration=1, trial = 1)*	Lifts cup to mouth by handle using one hand with pincer grasp	Lifts cup to mouth using one hand with full hand grasp	Lifts cup to mouth using both hands – full grasp	Touches cup but does not lift it or no attempt	Unable to assess
13	Feeds doll when requested to. Examiner says, "Can you feed dolly some tea?"<br>(Trials = 2, demonstration = 1 if not spontaneous on first attempt)	Spontaneously	After 1 demonstration	Partial attempt after 1 demonstr at-ion	No attempt	Unable to assess
14	Matches shapes in puzzle (objects in 1 line)<br>*(Trials = 5, demonstration = 0)*	All shapes in ≤3 trials	All shapes in 4-5 trials	1 or 2 shapes	No attempt	Unable to asses
15	Matches shapes on rotated puzzle (objects in 1 line)<br>*(Trials = 5, demonstration = 0)*	All shapes in ≤3 trials	All shapes in 4-5 trials	1 or 2 shapes	No attempt	Unable to asses
16	Unscrews lid of box to retrieve raisins<br>*(Trials = 2, demonstration=1 if first attempt unsuccessful)*	Unscrews lid in first attempt	Unscrews lid after demonstration	Attempts but unsuccess -ful	No attempt	Unable to asses
17	Puts a raisin precisely inside a small opening in a box<br>*(Trials =1, demonstration = 1, test both hands)*	Precise release of raisin into box with each hand	Clumsy release, raisin falls out of bottle with one or more hand	Attempts but unsuccess -ful release with one or more hand	No attempt	Unable to assess
18	Thumb-finger tip grasp – grasps raisin between thumb and index finger	Precisely grasps	Clumsily grasps	Grasps	Tries to	Unable to

	(Observed during item above, trials = 0, demonstration = 0)	raisin between thumb & index finger on both hands	raisin between thumb & index finger on either hand or both hands	the raisin with fingers on one or both hands	grasp raisin with palm or no attempt	assess
19	Child imitates 4 different consonant-vowel combinations e.g. 'dolly' 'baby' 'lorry' 'happy' 'cookie' *(Trials = 4; can ask child to repeat after mother)*	Imitates 3-4 consonant-vowel combinations correctly	Imitates 1-2 consonant-vowel combinations correctly	Imitates but not correctly	No attempt	Unable to assess
20	Responds to name by interrupting activity (scribbling) *(Trials = 3, demonstration = 0)*	Immediately on calling name when engaged in an activity	Only when mother calls child's name when child is involved in an activity	On repeated calling i.e. 2-3 trials	Does not respond to name	Unable to assess
21	Responds to 'no-no' e.g. stops reaching for an object (more crayons) when you say no-no *(Trials = 3, demonstration = 0)*	Child stops immediately (may or may not continue reaching after a few seconds)	Child stops on repeating no-no 2-3 times (may or may not continue reaching after a few seconds)	Child does not stop reaching but looks up	Child does not react to no-no	Unable to assess
22	Transfers ball from one hand to another (Trials =1, demonstration = 1)	Freely transfers ball from right to left and left to right hands	Transfers in one direction only	Unable to release ball during 1 or more transfers	No attempt	Unable to assess
23	Walks sideways with support *(Concurrent observation or maternal recall)*	Walks 3-4 steps with stepping movements	Walks 1-2 steps with stepping movements	Stands only (with/with -out	Stands momentaril y or no attempt	Unable to assess

				support)		
24	Stands alone for >3 seconds when placed in that position<br>*(Concurrent observation/trials = 1)*	Stands without support for >3 seconds	Stands without support for <1-3 seconds and then falls/reaches for support	Attempts but cannot stand at all without support	No attempt	Unable to assess
25	Walks alone<br>*(Concurrent observation/maternal recall)*	Walks 5 steps unsupported with stepping movements	Walks 1-3 steps unsupported with stepping movements	Walks forward supported by an adult	Cannot walk even if supported	Unable to assess
26	Raises self from sitting to standing position<br>*(Concurrent observation/maternal recall)*	Can stand up himself/herself with support and stay standing for 5 seconds (chair/table/adult's hand)	Can stand up himself/herself with support but cannot stay standing for 5 seconds (chair/table/adult's hand)	Tries to stand up with support but not able to stand up completely	Cannot stand up from sitting position at all,– or no attempt	Unable to assess
27	Two 2-4 syllable babble such as dada, mama but not specifically to any person or any thing *(Concurrent observation)*	Spontaneously	Mimics	1 syllable bable e.g. ma, da, pa	None	Unable to assess
28	Uses one meaningful word<br>*(Concurrent observation)*	Spontaneously, and in correct context	Spontaneously, but in incorrect context	Mimics on hearing	Does not use words	Unable to assess
29	Uses two meaningful words together<br>*(Concurrent observation)*	Spontaneously, and in correct context	Spontaneously, but in incorrect context	Mimics on hearing	Does not use words	Unable to assess
30	Combines word and gesture when asked	Combines word and gesture	Combines word and gesture	Combines word and	Does not combine	Unable to assess

	(Do not demonstrate, trials = 3, use different examples each time)	completely and appropriately	completely but inappropriately	gesture in-completely but in-appropriately	word and gesture	
A	What is the child's native (first) language? *(Caregiver report)*					
B	Does the child speak/understand any language other than his/her native language? *(Caregiver report)*					
C	Is the child exposed to any language other than his/her native language for more than 30 minutes each day on most days? *(Caregiver report)*					
D	What is the language in which the assessment is being conducted? *(Assessor reported)*					

**How often were the following behaviours observed in the child during the assessment?**				
31	Positive affect	Never or rarely	Some of the time	Most of the time
32	Exploration	Never or rarely	Some of the time	Most of the time
33	Ease of engagement	Never or rarely	Some of the time	Most of the time
34	Cooperativeness	Never or rarely	Some of the time	Most of the time
35	Adaptability to change	Never or rarely	Some of the time	Most of the time
36	Distractibility	Never or rarely	Some of the time	Most of the time
37	Negative Affect	Never or rarely	Some of the time	Most of the time
