# Supplementary material for: Construction and validation of the Oxford Neurodevelopment Assessment (OX-NDA) in 1-year-old Brazilian children
Source: BMC Pediatr. 2022 Dec 23;22:733. doi: 10.1186/s12887-022-03794-1 (PMC9783969; doi:10.1186/s12887-022-03794-1)
Supplement: Supplementary file 4 — Additional file 4: Fig. S4. [file 12887_2022_3794_MOESM4_ESM.pdf]

ADDITIONAL FILE 4

Figure S4 Bland-Altman Plots

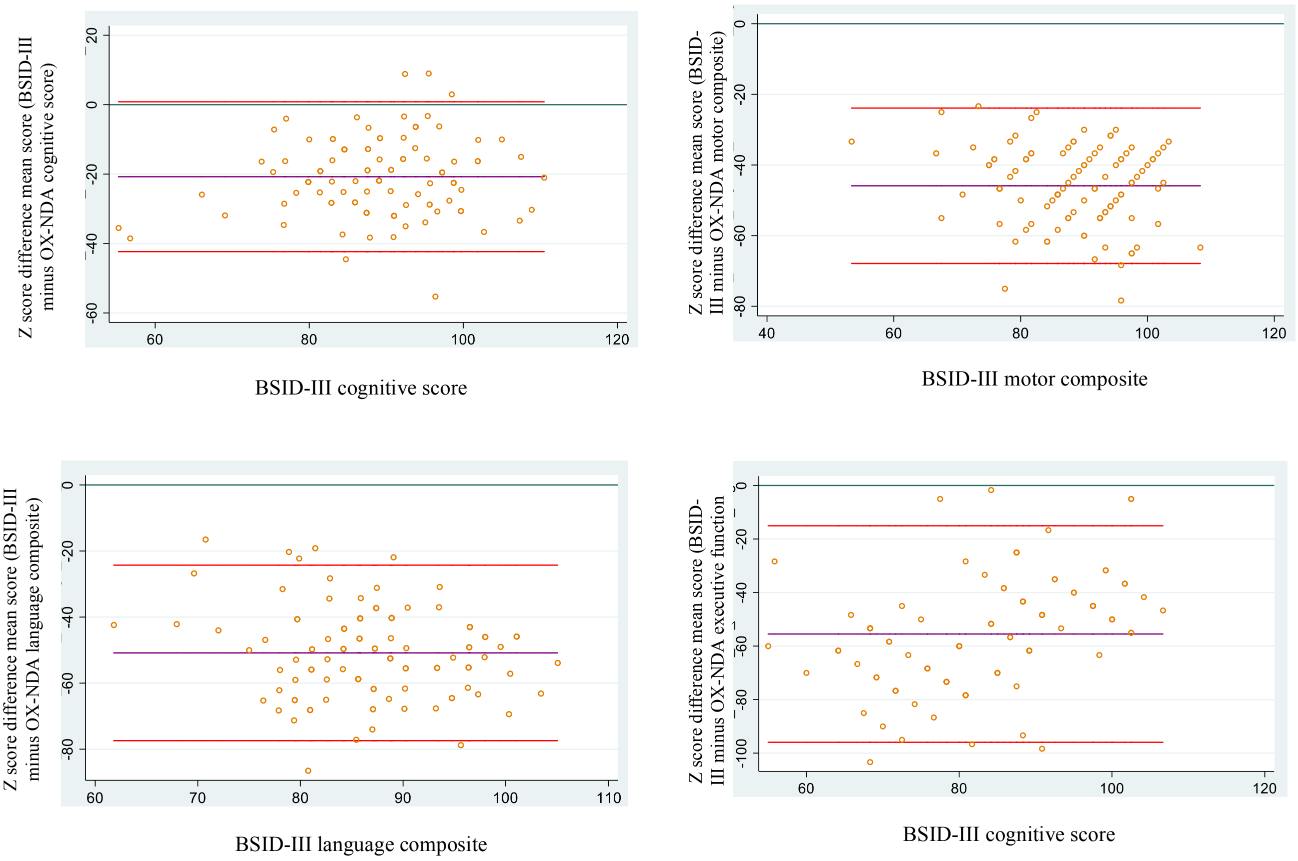

Y=0 line of perfect average agreement
Purple line = observed average agreement
Red lines = 95% limits of agreement
