# Supplementary material for: Construction and validation of the Oxford Neurodevelopment Assessment (OX-NDA) in 1-year-old Brazilian children
Source: BMC Pediatr. 2022 Dec 23;22:733. doi: 10.1186/s12887-022-03794-1 (PMC9783969; doi:10.1186/s12887-022-03794-1)
Supplement: Supplementary file 5 — Additional file 5: Table S5. [file 12887_2022_3794_MOESM5_ESM.pdf]

ADDITIONAL FILE 5

Table S5 Receiver Operating Characteristics of OX-NDA Domain Scores for Predicting Moderate-to-Severe Delay on the BSID-III (BSID-III composite score 70-85)

OX-NDA domain score threshold	Sensitivity	Specificity	PPV	NPV	LR+	LR-	Accuracy
**Cognition**							
≤53	0.94	0.50	0.98	0.25	1.88	0.12	0.72
≤54	0.88	0.50	0.98	0.14	1.76	0.24	0.69
≤56	0.84	0.75	0.99	0.16	3.36	0.21	0.80
≤57	0.79	0.75	0.99	0.13	3.16	0.28	0.77
≤59	0.76	0.75	0.98	0.11	3.04	0.32	0.76
≤60	0.76	0.75	0.98	0.11	3.04	0.32	0.76
≤62	0.60	1.00	1.00	0.009	.	0.40	0.80
≤64	0.56	1.00	1.00	0.008	.	0.44	0.78
**Motor**							
≤70	0.85	0.70	0.96	0.33	2.84	0.21	0.78
≤73	0.73	0.80	0.97	0.24	3.67	0.33	0.77
≤76	0.66	1.00	1.00	0.24	.	0.34	0.83
≤79	0.51	1.00	1.00	0.18	.	0.49	0.76
≤82	0.41	1.00	1.00	0.15	.	0.59	0.71
**Language**							
≤57	0.36	0.33	0.95	0.01	0.54	1.93	0.35
≤60	0.43	0.33	0.96	0.02	0.64	1.72	0.38
≤63	0.57	0	0.95	0	0.57	0	0.29
≤66	0.68	0	0.96	0	0.68	0	0.34
≤69	0.80	0	0.96	0	0.80	0	0.40
≤72	0.88	0	0.97	0	0.88	0	0.44

OX-NDA: The Oxford Neurodevelopment Assessment; PPV: Positive predictive value; NPV: Negative predictive value; LR+: Positive likelihood ratio; LR-: Negative likelihood ratio.
Shaded rows represent selected thresholds.
